# Opium Must Not Be Prohibited

**Published:** 1919-09

**Authors:** 


### OPIUM MUST NOT BE PROHIBITED

Dr. John Dill Robertson, Chicago's Commissioner of Health, has reported, in the daily papers, the result of a six-months' experiment conducted in the city's Tuberculosis Sanatorium, in doing without the use of opium or its derivatives; and he draws the conclusion that the use of opiates is, without exception, unnecessary. He urges that the medical profession ought to join voluntarily in a movement designed to prohibit the importation and sale of all the habit-forming drugs. In other words, although the chief object of medical science is to alleviate human suffering, it is proposed to deprive ourselves of our most reliable and efficient instrument to this end.

Because a lot of criminals and degenerates are abusing the use of morphine and cocaine, only to degrade themselves still further, the decent portion of humanity are to be forced to deny themselves the relief of pain caused by accident or disease, no matter how agonizing. Incidentally, the doctor asserts that there are 50,000 drug-addicts in Chicago. This is in line with the assertion lately made by the liquor-interest propagandists, that there are in the United States a million drug-addicts, and that prohibition would increase their number tenfold. Somehow, these assertions bring to mind the famous prediction, some two years ago, of a certain silver-tongued orator, that, if this country were threatened by a foreign foe, a million men would spring to arms between sunrise and sunset.

On a subject of such importance, it would seem fitting that extravagant assertions should be abandoned for uncontrovertible facts. Dr. McNamara reports that, in the Cook

County jail, in the six years from 1913 to 1919, out of 57,054 prisoners, only 755 were habitual drug-users, that is 1 in 75. It is a fact that the percentage of drug-users is much higher among criminals than among the community at large.

On this aspect of the case, the annual report of Surgeon-General Gorgas throws an interesting light. Out of 894,000 soldiers examined by the army-surgeons, only 403 were rejected as drug-users, that is, 1 in 2,218. It seems fair to assume that the proportion of addicts in the community at large would be somewhere near the same proportion, since these soldiers came from every part of the country. Taking our population at 100,000,000, that would give a little more than 45,000 drug-users in the United States. Then, if the population of Chicago is, in round numbers, 3,000,000, the same proportion would give the city only 1,350 addicts.

It is well enough to be philanthropic and self-sacrificing, for the sake of the unfortunate, but most of us will decline to go to the extent of denying relief to legitimate and unavoidable suffering for the sake of coddling the criminal classes.

Even if it were possible to make consumptives endure their sufferings without the relief afforded by morphine or heroin, that does not prove it either wise or necessary to deny them this relief. Doctor Robertson admits that during the first three days of the experiment the sanatorium was almost a bedlam. No wonder! He asserts that now opiates are not missed there. It would be interesting, indeed, to know just what the patients say about it. It is true that many a consumptive in the world's history has gone to his grave without the relief that morphine might have afforded him; but, was he any better off for having endured that suffering? Does not humanity demand that we make that dreadful period of hopeless misery as easy as possible?

Besides, there are other forms of pain that demand relief far more urgently than do the pangs of tuberculosis. What about the agonies of gallstone colic or renal colic or of the mangled and crushed bodies of the railway accident or of the battlefield? Indeed, it seems almost unbelievable that any intelligent person would seriously advocate a law that would render impossible the relieving of such suffering.

